# Losses in the Veterinary World

**Published:** 1898-02

**Authors:** 


### EDITORIAL.

### LOSSES IN THE VETERINARY WORLD.

The veterinary world loses, in the deaths of Prof. Finlay Dun, of Great Britain, Editor Lanzilotti Buonsanti, of Italy, and Prof. Charles Cornevin, of France, three able exponents of veterinary science.

In the therapeutic world the name of Finlay Dun is a very familiar one wherever the English language is read or spoken. Perhaps no English veterinary work has had so wide a sale as has his book on veterinary medicine; accepted as an authority, and covering the widest range of useful medicinal preparations, it has done much to advance veterinary science.

Dr. Buonsanti, as founder and director of one of the leading Italian veterinary periodicals and a writer and contributor to veterinary literature, made for himself a recognized place in the scientific world of comparative medicine, and added much to the progress of medical science and its recognition in his own country.

Prof. Cornevin was an honored instructor in the veterinary school at Lyons, France, where for many years he had endeared himself to a large number of students, and afterward practitioners, and these will all learn with sadness of his death and better appreciate his valued instruction as years go by. Prof. Cornevin was a contributor to many foregin journals, and recognition as an able writer was very generally accorded him.

